# Blood biomarkers and cognitive status in aged common marmosets

**DOI:** 10.1002/alz.091507

**Published:** 2025-01-09

**Authors:** Anusha Bharadwaj, Emily S. Rothwell, Carmen Freire‐Cobo, Stephanie L. Padilla, Patrick R Hof, Agnès Lacreuse

**Affiliations:** ^1^ University of Massachusetts‐Amherst, Amherst, MA USA; ^2^ University of Pittsburgh School of Medicine, Pittsburgh, PA USA; ^3^ Icahn School of Medicine at Mount Sinai, New York, NY USA

## Abstract

**Background:**

Nonhuman primates are arguably the best models for Alzheimer’s disease (AD), due to close similarities with humans in physiology and behavior, brain structure and function, and aging patterns. The common marmoset (Callithrix jacchus) is particularly valuable because it has the shortest lifespan of all anthropoids (10‐12 years), exhibits sex differences in age‐related cognitive decline and develops AD‐like pathology with age. In the present study, we investigated blood‐based biomarkers in male and female marmosets with cognitive and neuropathological assessments.

**Method:**

We analyzed the concentrations of several AD biomarkers from blood samples of male and female marmosets (n = 13, age range 6.8‐9.36 years) characterized for age‐related cognitive decline and neuropathology. AD biomarker concentrations were measured using the commercially available S‐PLEX neurology panel 1 kit (NHP) from Mesoscale Discovery. The kit, designed for GFAP, NfL, and tau detection, employs electrochemiluminescence to discern femtogram concentrations. Similar to ELISA, the emitted light intensity in S‐PLEX assays directly correlates with analyte amounts in the sample. Log‐transformed biomarker data were subjected to analyses of variance, with Sex and Cognitive Status as factors, and Age as a covariate.

**Result:**

LogGFAP concentrations were significantly higher in cognitively impaired (F (1, 8) = 15.50, p < .01) than unimpaired animals. Other biomarkers did not differ significantly based on cognitive status. All biomarkers exhibited non‐significant Sex effects (all ps < .10), suggesting higher levels in males. A non‐significant Sex x Cognitive Status interaction for LogGFAP (F (1, 8) = 4.12, p < .08) indicated higher levels in males among the impaired group. Although we found no significant association between blood biomarkers and neuropathological data, substantial correlations were noted within the impaired group. Analyses with a larger sample size are in progress.

**Conclusion:**

Alterations in biomarker concentrations serve as early indicators of cognitive decline, preceding AD diagnosis. Elevated blood GFAP, indicative of inflammation, differentiated impaired and non‐impaired aged marmosets, with sex influencing biomarker levels, predominantly higher in males. The absence of significant correlations between blood biomarkers and neuropathology necessitates further validation with a larger subject cohort.

Supported by NIH R01 AG046266 and SAGA 23

